# Characterization and complete genomic sequence of a novel phage BUCT805 infecting *Serratia marcescens* and its anti-biofilm activities

**DOI:** 10.1128/spectrum.03119-25

**Published:** 2026-02-23

**Authors:** Yuxuan Liu, Lefei Zhang, Yang Li, Lei Cao, Jianwei Zhang, Yigang Tong, Mengzhe Li

**Affiliations:** 1 College of Chemical Engineering, Beijing University of Chemical Technology 47832, Beijing, China; 2 BAICSM, State Key Laboratory of Green Biomanufacturing, College of Life Science and Technology, Beijing University of Chemical Technology 47832, Beijing, China; 3 College of Humanities and Law, Beijing University of Chemical Technology 47832, Beijing, China; Wannan Medical College, Wuhu, Anhui, China

**Keywords:** *Serratia marcescens*, characterization, genomic analysis, phage disinfection, biofilm removal

## Abstract

*Serratia marcescens* (*S. marcescens*) is an opportunistic pathogen commonly found in the environment and is capable of causing nosocomial and various severe infections. Bacteriophages (phages), as safe and eco-friendly natural antibacterial agents, hold promise for eradicating *S. marcescens* and its biofilms in hospital settings. In this study, we report a novel phage, BUCT805, isolated from wastewater, which effectively lysed *S. marcescens*, and we provide a detailed analysis of its physiological properties and genomic characteristics. BUCT805 was classified within the kingdom *Heunggongvirae* and the phylum *Uroviricota*. It formed clear plaques with a diameter of approximately 3.36 ± 0.63 mm, featuring a transparent center surrounded by a halo. The optimal multiplicity of infection for BUCT805 was 0.1, with a burst size of 338 ± 17 PFU per infected cell (*n* = 3), and the phage exhibited robust stability across a wide range of temperatures and pH levels. The genome of phage BUCT805 was composed of double-stranded DNA with a total length of 42,067 bp and a G+C content of 47%, exhibiting the highest sequence similarity to *Serratia* phage vB_SmaS_Serratianator with a query coverage of 88%. Importantly, no known antibiotic resistance or virulence genes were identified in the genome, and 40.32% of its open reading frames were annotated as functional proteins. Although the efficiency of biofilm removal by BUCT805 varied among different bacterial strains, it demonstrated significant biofilm eradication effects at high titers (10^9^ PFU/mL) against all tested strains. Overall, our findings supported the potential of phage BUCT805 as a promising candidate for the removal of *S. marcescens* biofilms from environmental settings.

**IMPORTANCE:**

Hospital surfaces could harbor *Serratia marcescens*, a resilient bacterium that forms protective biofilms and causes hospital-acquired infections (HAIs). We isolated and fully characterized BUCT805, a novel phage that specifically targeted *S. marcescens* and effectively removed its biofilms on plastic surfaces. BUCT805 was highly stable across a broad range of temperatures and pH, exhibited a high burst size, and carried no known antibiotic-resistance or toxin genes, supporting its safety for environmental applications. Phage BUCT805 had the potential to remove biofilms in the environment, thereby reducing the risk of HAI and providing an additional option for controlling *S. marcescens* and its biofilms in clinical settings.

## INTRODUCTION

Hospital environments serve as reservoirs for pathogenic bacteria and antibiotic resistance genes, significantly contributing to hospital-acquired infections (HAIs) (1–3). *Serratia marcescens* (*S. marcescens*), a gram-negative opportunistic pathogen commonly found in the environment, is prone to spreading among immunocompromised populations, particularly in intensive care units and neonatal intensive care units (4–6). *S. marcescens* exhibits resistance to multiple antibiotics and can cause severe diseases such as meningitis, pneumonia, endocarditis, and cellulitis (7–12). Under the regulation of quorum-sensing (QS) systems, *S. marcescens* is capable of forming biofilms, which further enhance its environmental adaptability, transmissibility, and resistance to antibiotics (13, 14). Moreover, a 6-year study revealed that among patients testing positive for *Serratia*, 92% were infected with *S. marcescens*, with most isolates originating from the community (15). The widespread use of antibiotics has driven an increase in both the diversity and abundance of antimicrobial resistance genes in *S. marcescens*, creating significant public health challenges (16).

Bacteriophages (phages), viruses that specifically infect bacteria, are the most abundant microorganisms in nature and represent a powerful tool against antibiotic-resistant bacteria (17, 18). Although phage therapy was initially used as an antibacterial strategy, its clinical application declined with the advent of antibiotics, only to be revived recently due to the global rise in antibiotic resistance (19). Due to their high specificity, phages can selectively target resistant bacteria and their biofilms without adversely affecting beneficial microbiota. Additionally, the combined use of phages and antibiotics has demonstrated potential in enhancing antibiotic efficacy and reducing resistance (20). With ongoing advancements in phage therapy, their applications in water treatment, food processing, aquaculture, and environmental disinfection are gradually being realized (21–26). Previous studies have leveraged the unique ability of phages to lyse host bacteria and disrupt biofilms, utilizing them as biocontrol agents alongside chemical disinfectants to eliminate environmental pathogens (27, 28). Notably, the use of phage cocktails in conjunction with chemical disinfectants can reduce the required dosages of these chemicals, thereby mitigating potential environmental and health risks (29). Thus, the rapid isolation and characterization of novel phages, along with the continuous expansion of phage libraries, are critical for advancing phage applications in clinical therapy and environmental disinfection.

In this study, we isolated a novel phage, BUCT805, and characterized its physiological and genomic properties. We also demonstrated its efficacy in eradicating *S. marcescens* biofilms on plastic surfaces, highlighting its potential for environmental disinfection.

## MATERIALS AND METHODS

### Bacteria and their incubation

*S. marcescens* strains used in this study were obtained from the Tong Lab collection and included both environmental isolates and clinical strains. All strains were stored at −80°C in 50% (vol/vol) glycerol in our laboratory. Initially, the strains were streaked onto Luria-Bertani (LB) agar plates and incubated at 37°C for 12 h. Subsequently, single colonies were inoculated and incubated in LB broth at 37°C for 12 h with shaking to reach the exponential growth phase.

### Phage isolation and purification

Phage was isolated from sewage samples using *S. marcescens* strain SM04 as the host. Briefly, 15 mL of sewage was centrifuged at 11,000 × *g* for 10 min, and the supernatant was filtered through a 0.22 μm membrane. The filtrate was then mixed with strain SM04 in LB broth and incubated at 37°C with shaking at 180 rpm for 24 h (30). After incubation, the mixture was centrifuged again at 11,000 × *g* for 10 min, and the supernatant was filtered through a 0.22 μm membrane. A volume of 100 μL of the filtered supernatant and 100 μL of an overnight culture of SM04 were mixed with LB soft agar and poured onto LB agar plates before solidification. The plates were incubated at 37°C for 12 h. Individual plaques were picked and subjected to three rounds of purification by the same procedure.

To prepare a high-titer phage solution, the purified phage was mixed with a 30% sucrose solution at a ratio of 4:1, centrifuged at 30,000 × *g* for 2 h at 4°C, and the supernatant was discarded. The resulting phage pellet was resuspended in 200 μL phosphate-buffered saline (PBS) to generate a concentrated phage stock (31).

### Transmission electron microscopy (TEM)

A 50 μL aliquot of the purified BUCT805 phage suspension (10^10^ PFU/mL) was applied onto a carbon-coated copper grid and allowed to air dry at room temperature for 30 min. After removing excess liquid, the grid was negatively stained with 2% phosphotungstic acid (32). Once fully air-dried at room temperature, the morphological characteristics of phage BUCT805 were observed using a transmission electron microscope (JEM-1400EX, JEOL, Tokyo, Japan) at 100 kV.

### Host range

To determine the host range of BUCT805, a panel of 20 *S*. *marcescens* strains from our bacterial collection was tested. Each strain was cultured in LB broth at 37°C with shaking (180 rpm) for 8 h. Then, 200 μL of the bacterial culture was mixed with LB soft agar and overlaid onto LB agar plates. 2 µL of BUCT805 phage suspensions with different titers (10^3^, 10^4^, 10^5^, 10^6^, 10^7^, and 10^8^ PFU/mL) were spotted onto the surface of the soft agar layer, with an equal volume of PBS used as the negative control. Plates were incubated overnight at 37°C, and the presence of plaques was observed to evaluate phage infectivity.

### Optimal multiplicity of infection (MOI) and one-step growth curve

To determine the optimal MOI, 100 μL of phage suspensions at different titers were mixed with 100 μL of the bacterial suspension (10^8^ CFU/mL) to achieve MOI values of 10, 1, 0.1, 0.01, 0.001, and 0.0001. Each mixture was subsequently added to 5 mL of LB broth and incubated at 37°C with shaking at 180 rpm for 12 h. After incubation, 1 mL of the mixture was centrifuged at 11,000 × *g* for 3 min, and the supernatant was filtered through a 0.22 μm membrane. Phage titer was then determined. This experiment was repeated three times, and the MOI resulting in the highest titer was considered the optimal MOI.

To analyze the latent period, burst time, and burst size of phage BUCT805, a one-step growth curve was constructed. 500 µL of *S. marcescens* SM04 (5 × 10^8^ CFU/mL) was mixed with 500 µL of phage BUCT805 (5 × 10^7^ PFU/mL). The mixture was incubated for 10 min to allow for adsorption. The mixture was then centrifuged at 11,000 × *g* for 2 min, and the supernatant was discarded. The pellet was resuspended in PBS, and the washing process was repeated two times. Subsequently, 1 mL of the washed mixture was transferred to 49 mL of LB broth and incubated at 37°C with shaking (220 rpm). Samples were taken at intervals over the course of 3 h, with each sample being filtered through a 0.22 μm membrane. Phage titer was measured using the soft agar overlay method. This experiment was repeated three times, and the average phage titer was calculated.

### Temperature stability and pH stability

To assess the stability of phage BUCT805 under different temperature conditions, 1 mL of purified phage BUCT805 (2.5 × 10^10^ PFU/mL) was transferred to a 1.5 mL centrifuge tube and incubated at various temperatures for 2 h. Phage titer was then determined using the soft agar overlay method. Similarly, to evaluate the stability of phage BUCT805 under different pH conditions, the phage (2.5 × 10^10^ PFU/mL) was incubated in buffers with different pH values at 37°C for 2 h. The phage titer was measured using the soft agar overlay method. Each experiment was repeated three times, and the average value was calculated.

### Phage DNA sequencing and bioinformatics analysis

Phage DNA was extracted using the Phage Genomic DNA Extraction Kit (LEAGENE, Beijing) according to the manufacturer’s instructions, employing the PEG precipitation method for DNA isolation (33). The DNA was then subjected to next-generation sequencing on the Illumina NovaSeq platform using the NEBNext Ultra II FS DNA Library Prep Kit (30). Raw data were quality controlled using Trimmomatic 0.36 to remove low-quality reads, followed by assembly with SPAdes v3.13.0. The open reading frames (ORFs) of the phage were predicted using the RAST tool (https://rast.nmpdr.org/, accessed on 3 May 2025), and the functional annotation of the ORFs was performed using BLASTp from the National Center for Biotechnology Information (NCBI, https://blast.ncbi.nlm.nih.gov/Blast.cgi, accessed on 3 May 2025). To construct a proteomic tree of the phage, the assembled sequence was compared with the NCBI BLASTn results and analyzed using the online tool ViPTree (https://www.genome.jp/viptree/, accessed on 5 May 2025).

### Biofilm removal evaluation

200 µL of a bacterial suspension at a concentration of 10^6^ CFU/mL was added to each well of a 96-well plate and incubated at 37°C in the dark for 24 h to allow biofilm formation on the well surfaces (27). After incubation, the supernatant was removed, and the wells were slowly washed two times with 200 µL of PBS. Afterward, all liquid was removed, and the plate was allowed to air dry at room temperature for 1 h. Once fully dried, 200 µL of phage at titers ranging from 10^4^ CFU/mL to 10^9^ CFU/mL was added to wells containing pre-formed biofilms, with an equal volume of PBS serving as the control. The plate was then incubated at 37°C in the dark for 4 h. After treatment, the supernatant was removed, and the wells were gently washed two times with 200 µL of PBS. The plate was left to air dry at room temperature for 30 min. Subsequently, 200 µL of 0.1% crystal violet solution was added for staining for 15 min (34). After staining, excess dye was removed, and the wells were decolorized with 95% ethanol for 20 min. The optical density (OD) at 550 nm was measured to quantify biofilm formation (35). Each experiment was repeated three times, and the average value was calculated.

### Statistical analysis

All data were processed using GraphPad Prism 10.1.2. The MOI, temperature stability, and pH stability of the phage were analyzed using one-way analysis of variance (ANOVA) followed by Dunnett’s multiple comparisons test. The effects of the phage on biofilm removal were analyzed using a *t*-test. Each experiment was performed in triplicate, and the average value and standard deviation were calculated. Statistical significance was considered at *P* < 0.05.

## RESULTS AND DISCUSSION

### Morphological characteristics of phage BUCT805

**Fig 1:**
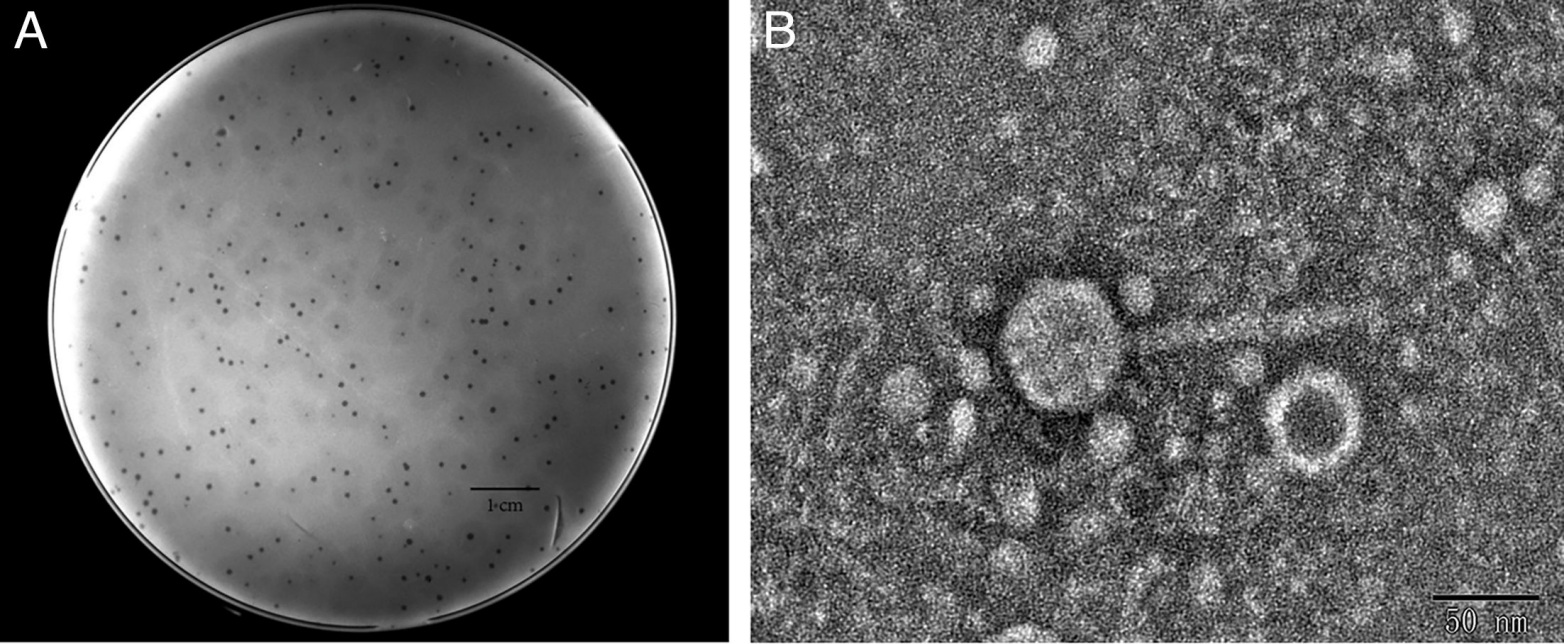
Morphological characteristics of phage BUCT805. (**A**) Plaques formed by phage BUCT805 on the *S. marcescens* SM04 bacterial lawn. (**B**) Transmission electron micrograph of phage BUCT805.

A novel bacteriophage, designated BUCT805, was isolated from sewage, with *S. marcescens* SM04 serving as the host. Phage BUCT805 formed clear plaques on the SM04 bacterial lawn, with an average plaque diameter of approximately 3.36 ± 0.63 mm (*n* = 10; Fig. 1A). The plaques exhibited a transparent center with a regular shape, surrounded by a translucent halo. TEM images revealed that the head of phage BUCT805 had an average diameter of approximately 53.89 ± 1.91 nm, while the tail had an average length of approximately 103.27 ± 2.39 nm (*n* = 10; Fig. 1B).

### Host range

The host range of phages is a crucial factor influencing their therapeutic efficacy and application in various fields (36). To assess the host range of phage BUCT805, we selected 20 *S*. *marcescens* strains derived from different sources (clinical and environmental) and evaluated the ability of phage BUCT805 to lyse these strains. The results showed that phage BUCT805 could lyse 50% of the selected strains. In addition to the original host strain SM04, BUCT805 also formed clear, well-defined plaques on the bacterial lawns of nine other *S. marcescens* strains (1219, 1261, 1413, 2860, 2861, 2862, 2863, 2864, and 2865) (Table 1). Compared with previous studies, phage BUCT805 exhibited a broader host range than *S. marcescens* phage ϕIF3, which infected only 2 of the 39 tested strains, and phage UFV01, which was infectious solely to its original isolate (37, 38).

**TABLE 1:** Host range analysis of phage BUCT805 against 20 strains

Species	Strains	Susceptibility	Origin
*Serratia marcescens*	SM04	+	Tong Lab
	1219	+	Tong Lab
	1252	−	Tong Lab
	1261	+	Tong Lab
	1373	−	Tong Lab
	1402	−	Tong Lab
	1413	+	Tong Lab
	1471	−	Tong Lab
	1800	−	Tong Lab
	1863	−	Tong Lab
	2703	−	Tong Lab
	2860	+	Tong Lab
	2861	+	Tong Lab
	2862	+	Tong Lab
	2863	+	Tong Lab
	2864	+	Tong Lab
	2865	+	Tong Lab
	2866	−	Tong Lab
	2867	−	Tong Lab
	2868	−	Tong Lab

### Optimal MOI and one-step growth curve of phage BUCT805

**Fig 2:**
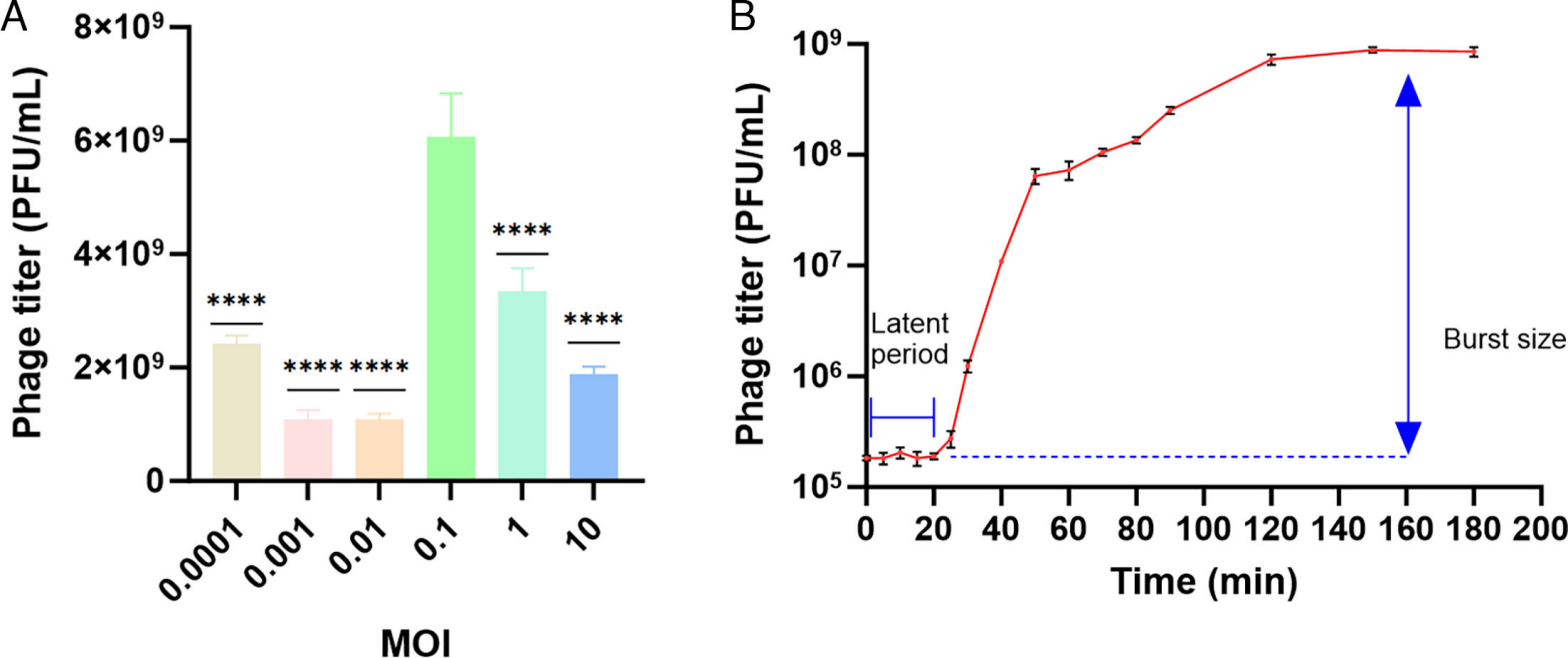
MOI and one-step growth curve of phage BUCT805. (**A**) MOI of phage BUCT805. (**B**) One-step growth curve of phage BUCT805. Time points were sampled densely at 0–30 min (every 5 min) and more sparsely thereafter to resolve rapid early dynamics. The data were analyzed using ANOVA followed by Dunnett’s multiple comparisons test. ****P < 0.0001.

To determine the optimal MOI of phage BUCT805, it was mixed with *S. marcescens* SM04 at different MOIs. When the MOI was 0.1, the average phage titer (6.06 × 10^9^ PFU/mL) was significantly higher than that in the other groups (Fig. 2A). Therefore, the optimal MOI for BUCT805 was determined to be 0.1. Subsequently, phage BUCT805 and *S. marcescens* SM04 were mixed at the optimal MOI to determine and plot the one-step growth curve. From 0 to 25 min, phage BUCT805 remained in the latent period, with no significant change in titer (Fig. 2B). After the latent period, phage titer rapidly increased from 2.73 × 10^5^ to 6.43 × 10^7^ PFU/mL between 25 and 50 min. After 50 min, the increase in phage titer gradually slowed, reaching a plateau and stabilizing at 120 min. The burst size was defined as the average number of phage particles released per infected cell. The burst size of phage BUCT805, calculated as (post-lysis titer − initial titer)/number of infected bacterial cells, was 338 ± 17 PFU per infected cell (*n* = 3). The number of infected bacterial cells was estimated based on the initial bacterial concentration and MOI. Compared with previous reports in which phage P-UFV01 released 157 viral particles per infected cell, phage BUCT805 exhibited a higher burst size, which suggested a strong bactericidal potential (38).

### Temperature and pH stability of phage BUCT805

**Fig 3:**
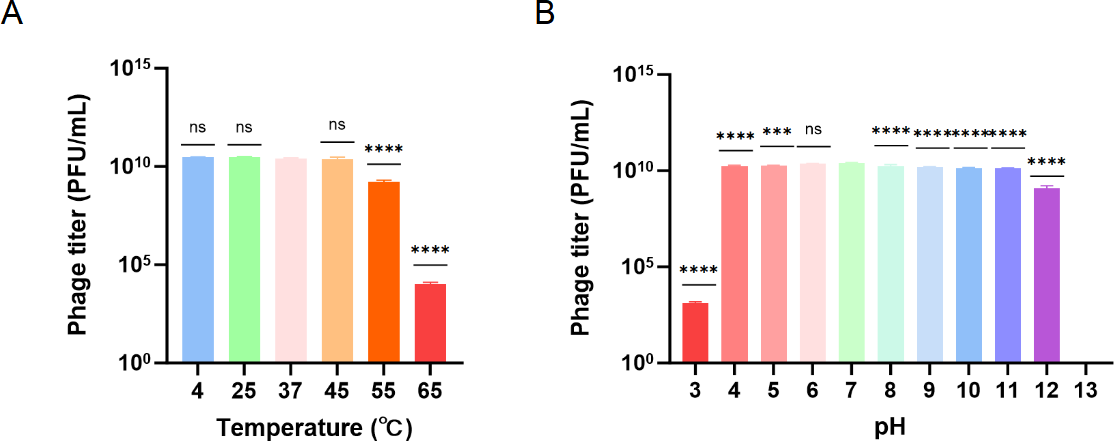
Temperature and pH stability of phage BUCT805. (**A**) Temperature stability of phage BUCT805. (**B**) pH stability of phage BUCT805. The data were analyzed using ANOVA followed by Dunnett’s multiple comparisons test. ***P < 0.001 and ****P < 0.0001; ns, not significant.

Phage BUCT805 maintained a relatively stable titer within the temperature range of 4°C to 45°C over a 2-h period, with no significant change observed (Fig. 3A). However, when the temperature was raised to 55°C, the titer decreased by approximately 10-fold compared to the titer at 37°C after 2 h. As the temperature increased further to 65°C, the titer dropped by approximately 2 × 10^6^-fold compared to the titer at 37°C over the same time period. These results indicated that phage BUCT805 exhibited considerable stability at room temperature, whereas its stability decreased under elevated temperature conditions. BUCT805 maintained a relatively stable titer during a 2-h incubation at pH 4–11, reaching a maximum titer of 2.51 × 10^10^ PFU/mL at pH 7 (Fig. 3B). At pH 3, the titer was approximately 2 × 10^7^-fold lower than at pH 7. After 2 h of incubation at pH 13, the titer dropped to 0 PFU/mL. Moreover, phage BUCT805 demonstrated superior pH stability compared with phage UFV01, although its stability under high-temperature conditions was inferior to that of phage KKP_3709 (39). These findings suggested that phage BUCT805 had poor stability in highly acidic or alkaline environments.

### Genomic features and ORF functional annotation of phage BUCT805

The genome of phage BUCT805 was 42,067 bp in length, with a G+C content of 47%. Analysis of the genome revealed that phage BUCT805 contained 62 ORFs, of which 25 (40.3%) were annotated as functional proteins, while the remaining 37 (59.7%) were classified as hypothetical proteins (Table 2).

**TABLE 2:** Features of the ORFs of phage BUCT805

ORF	Strand	Start	Stop	Length (AA)	Predicted protein function	Best-match BLASTp result	Query cover	*E*-values	Identity	Accession
1	+	4	87	27	Hypothetical protein	*Serratia* phage vB_SmaS_Bonzee	83%	9.1	50.00%	YP_010774122.1
2	+	96	674	192	Hypothetical protein	*Serratia* phage vB_SmaS_Bonzee	100%	2e−98	76.56%	YP_010774123.1
3	+	664	999	111	Hypothetical protein	*Serratia* phage vB_SmaS_Serratianator	100%	2e−55	75.89%	QPX74411.1
4	+	1,085	1,267	60	Hypothetical protein	*Serratia* phage vB_SmaS_Bonzee	100%	9e−28	81.67%	YP_010774125.1
5	+	1,270	1,767	165	Terminase small subunit	*Serratia* phage vB_SmaS_Bonzee	100%	8e−104	87.88%	YP_010774126.1
6	+	1,764	3,572	602	Terminase large subunit	*Serratia* phage vB_SmaS_Bonzee	100%	0.0	93.69%	YP_010774127.1
7	+	3,582	4,970	462	Portal protein	*Serratia* phage vB_SmaS_Bonzee	100%	0.0	87.64%	YP_010774128.1
8	+	4,951	5,610	219	Prohead protease	*Serratia* phage vB_SmaS_Bonzee	100%	5e−135	84.93%	YP_010774129.1
9	+	5,628	6,962	444	Major capsid protein	*Serratia* phage vB_SmaS_Bonzee	99%	0.0	86.85%	YP_010774130.1
10	+	7,038	7,391	117	Upper head-tail connector protein	*Serratia* phage vB_SmaS_Bonzee	100%	1e−63	78.63%	YP_010774131.1
11	+	7,393	7,752	119	Lower head-tail connector protein	*Serratia* phage vB_SmaS_Bonzee	99%	2e−74	89.83%	YP_010774132.1
12	+	7,766	8,311	181	Putative tail completion protein	*Serratia* phage vB_SmaS_Stoker	100%	4e−119	90.61%	YP_010774197.1
13	+	8,308	8,691	127	Tail terminator protein	*Serratia* phage vB_SmaS_Bonzee	100%	9e−77	87.40%	YP_010774134.1
14	+	8,704	9,417	237	Major tail tube protein	*Serratia* phage vB_SmaS_Bonzee	98%	8e−153	90.13%	YP_010774135.1
15	+	9,465	9,938	157	Hypothetical protein	*Serratia* phage vB_SmaS_Stoker	95%	3e−78	75.17%	YP_010774200.1
16	+	9,986	10,075	29	Hypothetical protein	*Serratia* phage vB_SmaS_Bonzee	100%	2e−08	86.21%	YP_010774137.1
17	+	10,085	12,331	748	Tail tape measure protein	*Serratia* phage vB_SmaS_Bonzee	100%	0.0	85.91%	YP_010774138.1
18	+	12,394	12,618	74	Hypothetical protein	*Serratia* phage vB_SmaS_Ulliraptor	91%	2e−14	52.94%	YP_010774274.1
19	+	12,684	12,854	56	Hypothetical protein	*Serratia* phage vB_SmaS_Serratianator	100%	1e−31	98.21%	QPX74395.1
20	+	12,851	12,964	37	Hypothetical protein	No hit	No hit	No hit	No hit	No hit
21	+	13,053	13,244	63	Hypothetical protein	*Serratia* phage vB_SmaS_Bonzee	100%	1e−25	74.60%	YP_010774143.1
22	−	13,920	13,294	208	Hypothetical protein	No hit	No hit	No hit	No hit	No hit
23	+	13,971	14,684	237	Tail tip complex protein	*Serratia* phage vB_SmaS_Stoker	100%	5e−153	85.65%	YP_010774209.1
24	+	14,681	15,463	260	Tail tip complex protein	*Serratia* phage vB_SmaS_Bonzee	100%	9e−148	75.77%	YP_010774147.1
25	+	15,456	16,067	203	Tail assembly protein	*Serratia* phage vB_SmaS_Bonzee	100%	2e−120	87.19%	YP_010774148.1
26	+	16,077	18,176	699	Tail spike protein	*Serratia* phage vB_SmaS_Niamh	100%	0.0	88.27%	UGO52992.1
27	+	18,179	18,952	257	Hypothetical protein	*Serratia* phage vB_SmaS_Bigdog	100%	1e−162	89.49%	YP_010774350.1
28	+	19,021	22,185	1,054	Tail tip protein	*Serratia* phage vB_SmaS_Bonzee	100%	0.0	85.66%	YP_010774151.1
29	+	22,305	22,784	159	Hypothetical protein	*Serratia* phage vB_SmaS_Bonzee	100%	2e−108	100.00%	YP_010774152.1
30	+	22,928	23,200	90	Hypothetical protein	Klebsiella phage vB_KmiS-Kmi2C	93%	3e−16	52.38%	UYE92018.1
31	−	23,508	23,224	94	Hypothetical protein	*Serratia* phage vB_SmaS_Ulliraptor	100%	3e−49	76.60%	YP_010774289.1
32	−	23,846	23,505	113	Hypothetical protein	*Serratia* phage vB_SmaS_Stoker	100%	4e−68	85.84%	YP_010774218.1
33	−	24,693	23,851	280	DNA-like exonuclease	*Serratia* phage vB_SmaS_Bonzee	100%	0.0	88.21%	YP_010774157.1
34	−	25,010	24,690	106	Hypothetical protein	*Serratia* phage vB_SmaS_Bigdog	98%	2e−42	67.31%	YP_010774357.1
35	−	25,378	25,061	105	Hypothetical protein	*Serratia* phage vB_SmaS_Bigdog	100%	6e−62	84.76%	YP_010774358.1
36	−	27,072	25,375	565	ATP-dependent helicase	*Serratia* phage vB_SmaS_Bonzee	100%	0.0	89.73%	YP_010774160.1
37	−	28,083	27,073	336	Exonuclease	*Serratia* phage vB_SmaS_Bonzee	97%	0.0	86.24%	YP_010774161.1
38	−	29,052	28,135	305	Hypothetical protein	*Serratia* phage vB_SmaS_Bonzee	89%	5e−112	73.88%	YP_010774162.1
39	−	29,758	29,066	230	Hypothetical protein	*Serratia* phage vB_SmaS_Stoker	100%	4e−160	93.04%	YP_010774225.1
40	+	29,837	30,070	77	Hypothetical protein	*Serratia* phage vB_SmaS_Ulliraptor	96%	2e−27	66.22%	YP_010774298.1
41	+	30,048	32,642	864	DNA primase/polymerase	*Serratia* phage vB_SmaS_Bonzee	100%	0.0	94.33%	YP_010774165.1
42	+	32,867	33,322	151	Hypothetical protein	*Serratia* phage vB_SmaS_Bigdog	98%	4e−71	70.27%	YP_010774365.1
43	+	33,303	33,521	72	Hypothetical protein	*Serratia* phage vB_SmaS_Bonzee	100%	3e−37	86.11%	YP_010774167.1
44	+	33,518	33,805	95	Hypothetical protein	*Serratia* phage vB_SmaS_Bonzee	100%	3e−33	63.16%	YP_010774168.1
45	−	34,098	33,904	64	Hypothetical protein	No hit	No hit	No hit	No hit	No hit
46	+	34,371	34,979	202	Hypothetical protein	*Serratia* phage vB_SmaS_Niamh	98%	2e−66	52.53%	UGO53014.1
47	+	35,226	35,372	48	Hypothetical protein	*Serratia* phage vB_SmaS_Bonzee	100%	3e−15	77.08%	YP_010774172.1
48	+	35,375	35,869	164	VRR-NUC domain-containing protein	*Serratia* phage vB_SmaS_Bonzee	100%	4e−103	85.98%	YP_010774173.1
49	+	35,916	36,308	130	Hypothetical protein	*Serratia* phage vB_SmaS_Niamh	97%	2e−60	74.60%	UGO53018.1
50	+	36,305	36,571	88	Holin	*Serratia* phage vB_SmaS_Bonzee	99%	2e−51	93.10%	YP_010774175.1
51	+	36,564	37,040	158	Lysozyme	*Serratia* phage vB_SmaS_Bonzee	100%	3e−89	79.75%	YP_010774176.1
52	+	37,053	37,322	89	I-spanin	*Serratia* phage vB_SmaS_Bigdog	100%	7e−34	80.90%	YP_010774376.1
53	+	37,748	38,020	90	Hypothetical protein	*Serratia* phage vB_SmaS_Stoker	100%	7e−50	81.11%	YP_010774242.1
54	+	38,023	38,373	116	HNH endonuclease	*Serratia* phage vB_SmaS_Bonzee	100%	2e−62	78.45%	YP_010774181.1
55	+	38,641	38,982	113	Hypothetical protein	*Serratia* phage vB_SmaS_Bonzee	89%	5e−06	33.66%	YP_010774114.1
56	+	38,990	39,319	109	Hypothetical protein	*Serratia* phage vB_SmaS_Stoker	100%	1e−53	74.31%	YP_010774245.1
57	+	39,312	39,659	115	Hypothetical proteinNot available	Not available	No hit	No hit	No hit	No hit
58	+	39,716	40,000	94	Hypothetical protein	*Serratia* phage vB_SmaS_Stoker	96%	6e−18	47.78%	YP_010774182.1
59	+	39,997	40,215	72	Hypothetical protein	Not available	No hit	No hit	No hit	No hit
60	+	40,362	41,159	265	Hypothetical protein	*Serratia* phage vB_SmaS_Bonzee	99%	2e−72	47.06%	YP_010774118.1
61	+	41,373	41,609	78	Hypothetical protein	*Serratia* phage vB_SmaS_Stoker	100%	9e−49	97.44%	YP_010774184.1
62	+	41,590	41,847	85	Hypothetical protein	*Serratia* phage vB_SmaS_Stoker	98%	2e-53	100.00%	YP_010774185.1

**Fig 4:**
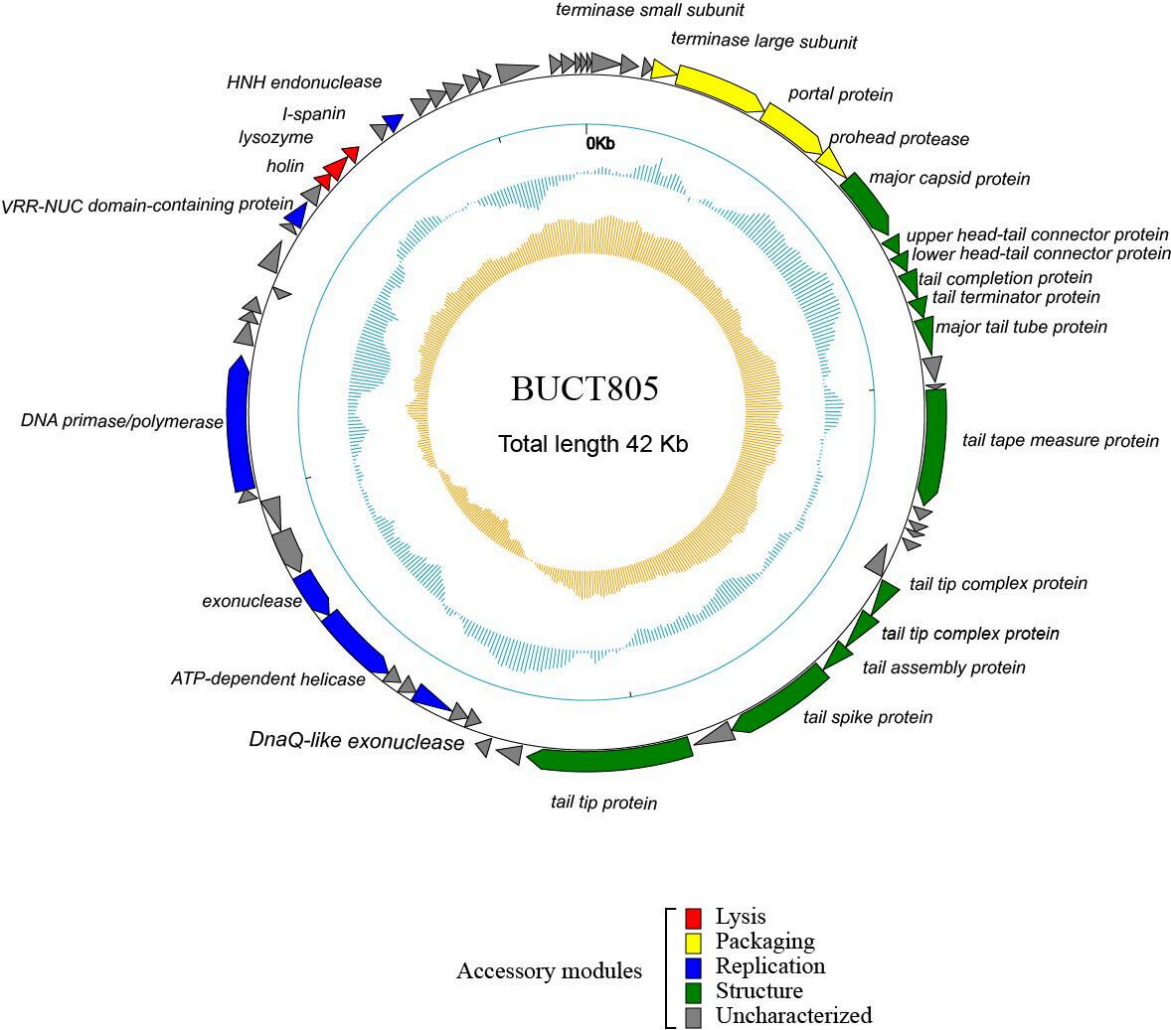
Genome map of phage BUCT805. The innermost yellow circle represents the G−C/G+C skew, with inward indicating values less than 0 and outward indicating values greater than 0. The middle blue circle represents the (G+C) mol% content, with inward indicating lower-than-average content and outward indicating higher-than-average content. The outermost circle corresponds to the annotation of ORF functions, with different colors representing distinct functional categories.

The functional proteins annotated in phage BUCT805 were classified into four categories based on their gene functions: Lysis, Packaging, Replication, and Structure. The analysis showed that proteins responsible for the packaging and tail structure were primarily located in the first half of the genome, while those involved in lysis and replication were mainly distributed in the latter half of the genome (Fig. 4). Among these, ORF5, ORF6, ORF7, and ORF8 are associated with packaging, while ORF33, ORF36, ORF37, ORF41, ORF48, and ORF54 were involved in replication. Additionally, ORF9, ORF10, ORF11, ORF12, ORF13, ORF14, ORF17, ORF23, ORF24, ORF25, ORF26, and ORF28 were related to the phage structure, and ORF50, ORF51, and ORF52 were associated with phage lysis.

Holin is a hydrophobic protein that forms pores in the cell membrane, thereby providing a pathway for endolysins to enter the cell and playing a crucial role in the lysis process (40). Most phages produce a two-component spanin complex to disrupt the outer membrane (41). Analysis of the BUCT805 genome revealed the concurrent presence of three lysis-related genes: holin (ORF50), lysozyme (ORF51), and I-spanin (ORF52). These proteins may act synergistically, contributing to an expanded host range and enhanced lytic capability. Investigating their synergistic mechanisms could offer novel insights for the design of engineered phages. Moreover, genomic analysis indicated that phage BUCT805 does not harbor any known resistance genes or virulence factors. Notably, hypothetical proteins account for 59.68% of the genome; further study of these proteins may provide fresh perspectives on phage biology.

### Proteomic tree of phage BUCT805

**Fig 5:**
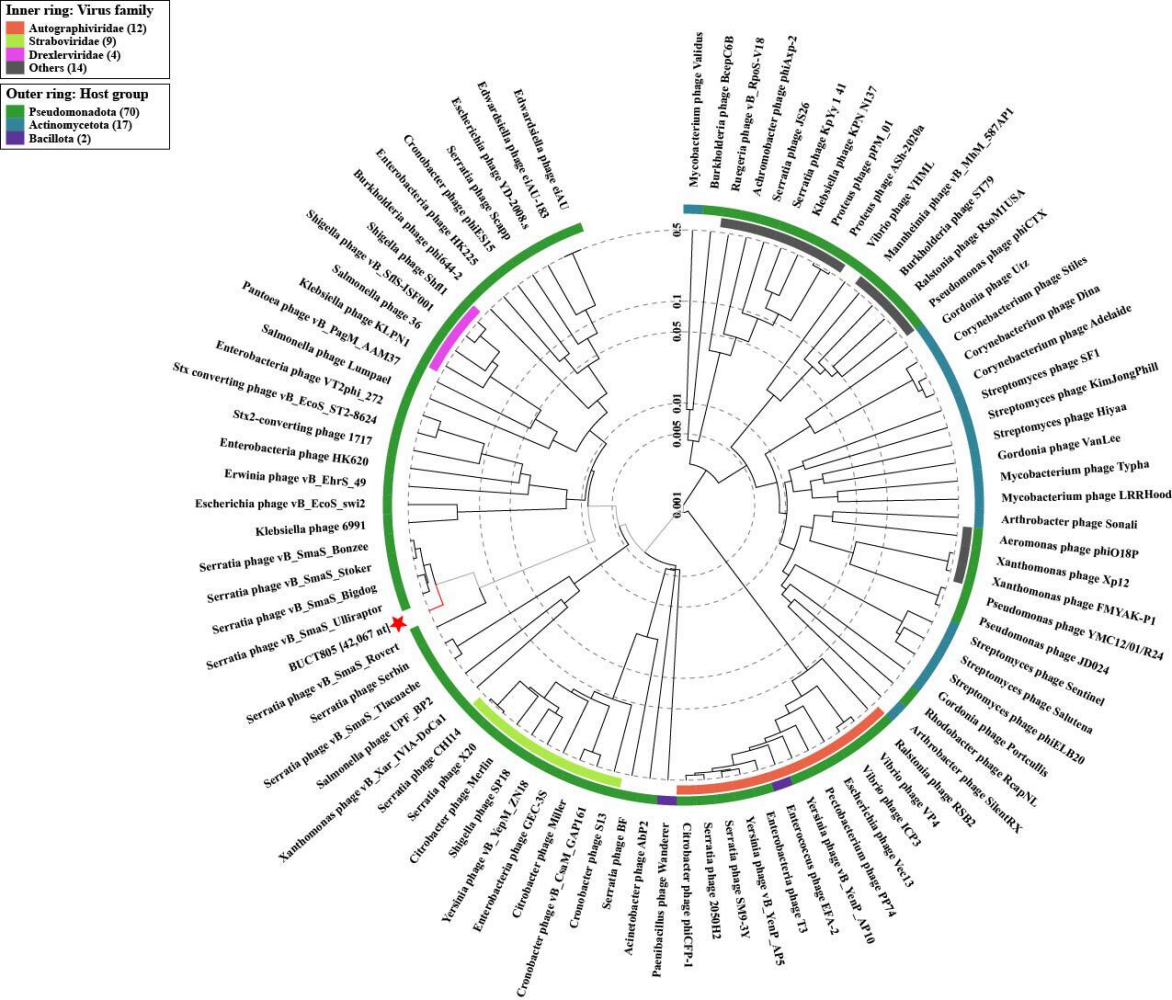
Proteomic tree of phage BUCT805.

To further investigate the evolutionary relationship between phage BUCT805 and other phages, a proteomic tree including 90 phages, with phage BUCT805 as one of the members, was constructed using the online tool ViPTree (38). The results showed that phage BUCT805 was more closely related to *Serratia* phage vB_SmaS_Ulliraptor (NC_074754) in terms of evolutionary relationship (Fig. 5). Additionally, a Blastn search revealed that phage BUCT805 shared the highest similarity with *Serratia* phage vB_SmaS_Serratianator, with a query coverage of 88% and a percent identity of 79.82%. Based on these findings, it was inferred that phage BUCT805 belonged to the realm *Duplodnaviria*, kingdom *Heunggongvirae*, phylum *Uroviricota*, and class *Caudoviricetes*.

### Biofilm removal efficiency of phage BUCT805

**Fig 6:**
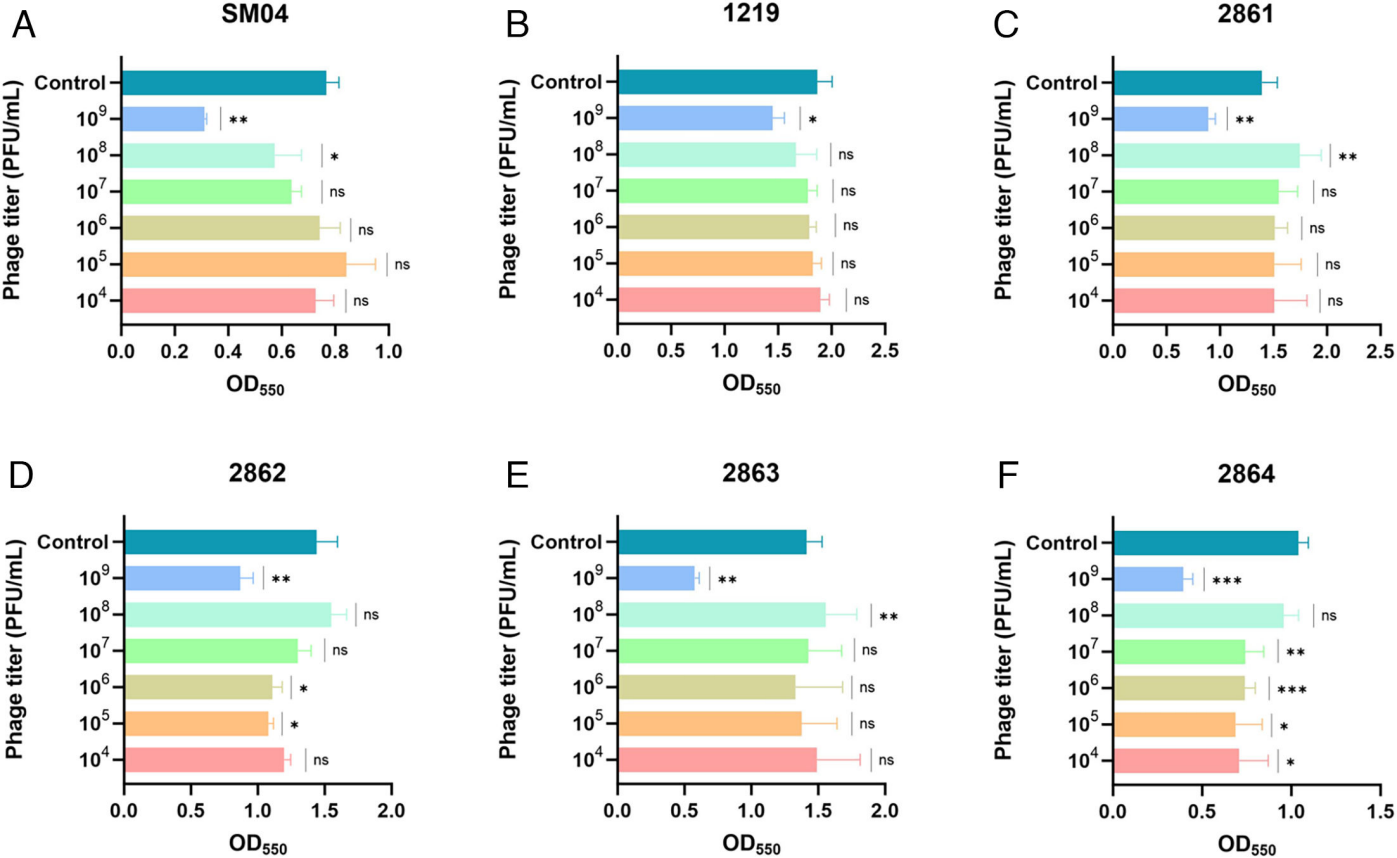
Biofilm removal efficiency of phage BUCT805. (**A–F**) The removal efficacy of phage at titers ranging from 10^9^ to 10^4^ PFU/mL against strains SM04, 1219, 2861, 2862, 2863 and 2864. The *t*-test was used to compare the control group and phage-treated group with different titers for the same bacterial strain. *P < 0.05, **P < 0.01, and ***P < 0.001; ns, not significant.

The ability of *S. marcescens* to form biofilms on catheters or implants increased the risk of HAIs (42). The results indicated that although phage BUCT805 was capable of removing *S. marcescens* biofilms, its efficacy varied among different strains. Specifically, a titer of 10⁹ PFU/mL proved to be optimal for biofilm removal in strains SM04, 2861, 2862, and 2863 (Fig. 6A, C, D and E), with removal efficiencies of 59.39%, 35.88%, 39.59%, and 59.08%, respectively. In contrast, phage BUCT805 showed the weakest biofilm removal against strain 1219, removing only approximately 22.34% of the biofilm within 4 h at a titer of 10^9^ PFU/mL (Fig. 6B). When the phage titer was reduced to between 10^8^ and 10^4^ PFU/mL, no significant biofilm removal was observed for strain 1219. Additionally, phage BUCT805 demonstrated the most pronounced biofilm removal against strain 2864, achieving a removal rate of 62.11% at a titer of 10^9^ PFU/mL within 4 h (Fig. 6F). In summary, phage BUCT805 exhibited the potential to remove *S. marcescens* biofilms from plastic surfaces.

Biofilms, composed of bacteria and extracellular polymers forming a self-produced hydrated matrix, enhance the colonization and dissemination potential of *S. marcescens* in hospital environments (43, 44). The presence of a halo around the plaques suggested that the phage might encode a depolymerase, which warranted further experimental verification (45). In this study, we demonstrated that phage BUCT805 can efficiently remove *S. marcescens* biofilms on plastic surfaces within 4 h. Although the biofilm removal efficacy of phage BUCT805 varied among different strains, our findings support its potential application in the disinfection of plastic surfaces; however, the measurement of biofilm biomass at 550 nm, instead of the optimal wavelength, due to equipment limitations, may have slightly affected the assay sensitivity.

### Conclusion

A novel phage, BUCT805, isolated from wastewater, is capable of lysing *S. marcescens* and effectively removing its biofilms. Phage BUCT805 demonstrated relatively good stability across different temperatures and pH values and exhibited a high burst size. Moreover, genomic analysis indicated that phage BUCT805 does not harbor any known resistance genes or virulence factors. Notably, hypothetical proteins account for 59.68% of the genome. Future research should further elucidate the functions of the hypothetical proteins encoded by the BUCT805 genome and explore combined treatment strategies involving phages, probiotics, and chemical disinfectants.

## Data Availability

The genome sequence of BUCT 805 has been submitted to GenBank with accession number PV277656.1.

## References

[1] Yang Q, Zhang M, Tu Z, Sun Y, Zhao B, Cheng Z, Chen L, Zhong Z, Ye Y, Xia Y. 2024. Department-specific patterns of bacterial communities and antibiotic resistance in hospital indoor environments. Appl Microbiol Biotechnol 108:487. doi:10.1007/s00253-024-13326-9 39412549 PMC11485044

[2] Tamsi NSF, Latif MT, Othman M, Abu Bakar FD, Yusof HM, Noraini NMR, Zahaba M, Sahani M. 2022. Antibiotic resistance of airborne bacterial populations in a hospital environment. Environ Monit Assess 194:629. doi:10.1007/s10661-022-10291-6 35918614

[3] Westover C, Rahmatulloev S, Danko D, Afshin EE, O’Hara NB, Ounit R, Bezdan D, Mason CE. 2023. Ozone disinfection for elimination of bacteria and degradation of Sars-CoV2 RNA for medical environments. Genes (Basel) 14:85. doi:10.3390/genes14010085 PMC9858956 36672826

[4] Caggiano G, Triggiano F, Diella G, Apollonio F, Lopuzzo M, Mosca A, Stolfa S, Pazzani C, Oliva M, Calia C, Laforgia N, Dalfino L, Barbuti G, Stefanizzi P, Minicucci AM, De Giglio O, Montagna MT. 2021. A possible outbreak by Serratia marcescens: genetic relatedness between clinical and environmental strains. Int J Environ Res Public Health 18:9814. doi:10.3390/ijerph18189814 34574734 PMC8472797

[5] Tavares-Carreon F, De Anda-Mora K, Rojas-Barrera IC, Andrade A. 2023. Serratia marcescens antibiotic resistance mechanisms of an opportunistic pathogen: a literature review. PeerJ 11:e14399. doi:10.7717/peerj.14399 36627920 PMC9826615

[6] Jones BL, Gorman LJ, Simpson J, Curran ET, McNamee S, Lucas C, Michie J, Platt DJ, Thakker B. 2000. An outbreak of Serratia marcescens in two neonatal intensive care units. J Hosp Infect 46:314–319. doi:10.1053/jhin.2000.0837 11170764

[7] Cooper R, Mills J. 1980. Serratia endocarditis. a follow-up report. Arch Intern Med 140:199–202. doi:10.1001/archinte.1980.00330140057018 6986128

[8] Balikian JP, Herman PG, Godleski JJ. 1980. Serratia pneumonia. Radiology 137:309–311. doi:10.1148/radiology.137.2.7001538 7001538

[9] Zivkovic Zaric R, Zaric M, Sekulic M, Zornic N, Nesic J, Rosic V, Vulovic T, Spasic M, Vuleta M, Jovanovic J, Jovanovic D, Jakovljevic S, Canovic P. 2023. Antimicrobial treatment of Serratia marcescens invasive infections: systematic review. Antibiotics (Basel) 12:367. doi:10.3390/antibiotics12020367 36830278 PMC9952094

[10] Mahlen SD. 2011. Serratia infections: from military experiments to current practice. Clin Microbiol Rev 24:755–791. doi:10.1128/CMR.00017-11 21976608 PMC3194826

[11] Fournier JB, Dabiri G, Thomas V, Skowron G, Carson P, Falanga V. 2016. Serratia marcescens bullous cellulitis in a splenectomized patient: a case report and review of the literature. Int J Low Extrem Wounds 15:161–168. doi:10.1177/1534734616636271 27079487

[12] Sannathimmappa MB, Nambiar V, Al Siyabi KHSH, Hussain AS, Shah YA, Marimuthu Y, Al-Maqbali S, Annamanedi M, Al-Risi ES, Aravindakshan R. 2024. Antimicrobial resistance pattern, predisposing factors, and outcome of Serratia infection in patients treated at a secondary-care hospital in oman: a 5-year retrospective study. Adv Biomed Res 13:101. doi:10.4103/abr.abr_381_23 39717260 PMC11665163

[13] Bakkiyaraj D, Sivasankar C, Pandian SK. 2012. Inhibition of quorum sensing regulated biofilm formation in Serratia marcescens causing nosocomial infections. Bioorganic & Medicinal Chemistry Letters 22:3089–3094. doi:10.1016/j.bmcl.2012.03.063 22487181

[14] Ray C, Shenoy AT, Orihuela CJ, González-Juarbe N. 2017. Killing of Serratia marcescens biofilms with chloramphenicol. Ann Clin Microbiol Antimicrob 16:19. doi:10.1186/s12941-017-0192-2 28356113 PMC5370475

[15] Laupland KB, Parkins MD, Gregson DB, Church DL, Ross T, Pitout JDD. 2008. Population-based laboratory surveillance for Serratia species isolates in a large Canadian health region. Eur J Clin Microbiol Infect Dis 27:89–95. doi:10.1007/s10096-007-0400-7 17960436

[16] Hou J, Mao DQ, Zhang YL, Huang RY, Li LY, Wang XL, Luo Y. 2022. Long-term spatiotemporal variation of antimicrobial resistance genes within the Serratia marcescens population and transmission of S. marcescens revealed by public whole-genome datasets. J Hazard Mater 423:127220. doi:10.1016/j.jhazmat.2021.127220 34844350

[17] Labrie SJ, Samson JE, Moineau S. 2010. Bacteriophage resistance mechanisms. Nat Rev Microbiol 8:317–327. doi:10.1038/nrmicro2315 20348932

[18] Hibstu Z, Belew H, Akelew Y, Mengist HM. 2022. Phage therapy: a different approach to fight bacterial infections. Biologics 16:173–186. doi:10.2147/BTT.S381237 36225325 PMC9550173

[19] Strathdee SA, Hatfull GF, Mutalik VK, Schooley RT. 2023. Phage therapy: from biological mechanisms to future directions. Cell 186:17–31. doi:10.1016/j.cell.2022.11.017 36608652 PMC9827498

[20] Cui L, Watanabe S, Miyanaga K, Kiga K, Sasahara T, Aiba Y, Tan X-E, Veeranarayanan S, Thitiananpakorn K, Nguyen HM, Wannigama DL. 2024. A comprehensive review on phage therapy and phage-based drug development. Antibiotics (Basel) 13:870. doi:10.3390/antibiotics13090870 39335043 PMC11428490

[21] Moye ZD, Woolston J, Sulakvelidze A. 2018. Bacteriophage applications for food production and processing. Viruses 10:205. doi:10.3390/v10040205 29671810 PMC5923499

[22] Brás A, Braz M, Martinho I, Duarte J, Pereira C, Almeida A. 2024. Effect of bacteriophages against biofilms of Escherichia coli on food processing surfaces. Microorganisms 12:2. doi:10.3390/microorganisms12020366 PMC10892694 38399770

[23] Garvey M. 2022. Bacteriophages and food production: biocontrol and bio-preservation options for food safety. Antibiotics (Basel) 11:10. doi:10.3390/antibiotics11101324 PMC9598955 36289982

[24] Abbas RZ, Alsayeqh AF, Aqib AI. 2022. Role of bacteriophages for optimized health and production of poultry. Animals (Basel) 12:23. doi:10.3390/ani12233378 PMC9736383 36496899

[25] Hegarty B. 2025. Making waves: intelligent phage cocktail design, a pathway to precise microbial control in water systems. Water Res 268:122594. doi:10.1016/j.watres.2024.122594 39405620

[26] Oliveira IM, Gomes IB, Simões LC, Simões M. 2024. A review of research advances on disinfection strategies for biofilm control in drinking water distribution systems. Water Res 253:121273. doi:10.1016/j.watres.2024.121273 38359597

[27] Stachler E, Kull A, Julian TR. 2021. Bacteriophage treatment before chemical disinfection can enhance removal of plastic-surface-associated Pseudomonas aeruginosa. Appl Environ Microbiol 87:e0098021. doi:10.1128/AEM.00980-21 34347517 PMC8478462

[28] Song J, Ruan H, Chen L, Jin Y, Zheng J, Wu R, Sun D. 2021. Potential of bacteriophages as disinfectants to control of Staphylococcus aureus biofilms. BMC Microbiol 21:57. doi:10.1186/s12866-021-02117-1 33607940 PMC7896381

[29] Xue YB, Gao Y, Guo MT, Zhang YM, Zhao GQ, Xia L, Ma JJ, Cheng YQ, Wang HG, Sun JH, Wang ZF, Yan YX. 2024. Phage cocktail superimposed disinfection: a ecological strategy for preventing pathogenic bacterial infections in dairy farms. Environ Res 252:118720. doi:10.1016/j.envres.2024.118720 38537740

[30] Han P, Pu M, Li Y, Fan H, Tong Y. 2023. Characterization of bacteriophage BUCT631 lytic for K1 Klebsiella pneumoniae and its therapeutic efficacy in Galleria mellonella larvae. Virol Sin 38:801–812. doi:10.1016/j.virs.2023.07.002 37419417 PMC10590696

[31] Han K, Dong Y, An X, Song L, Li M, Fan H, Tong Y. 2022. Potential application of a newly isolated phage BUCT609 infecting Stenotrophomonas maltophilia Front Microbiol 13:1001237. doi:10.3389/fmicb.2022.1001237 36478859 PMC9720304

[32] Domingo-Calap P, Beamud B, Mora-Quilis L, González-Candelas F, Sanjuán R. 2020. Isolation and characterization of two Klebsiella pneumoniae phages encoding divergent depolymerases. Int J Mol Sci 21:3160. doi:10.3390/ijms21093160 32365770 PMC7246685

[33] Xiao ZD, Hu XW, Chen JT, Xue MY, Zhang CJ, Jiang N, Liu XD, Fan YD, Kong XH, Zhou Y. 2024. Isolation and characterization of Bacillus cereus virulent phage CA1. Aquaculture 589:740989. doi:10.1016/j.aquaculture.2024.740989

[34] O’Toole GA. 2011. Microtiter dish biofilm formation assay. J Vis Exp 47:2437. doi:10.3791/2437 PMC3182663 21307833

[35] Chen Z, Yang Y, Li G, Huang Y, Luo Y, Le S. 2024. Effective elimination of bacteria on hard surfaces by the combined use of bacteriophages and chemical disinfectants. Microbiol Spectr 12:e0379723. doi:10.1128/spectrum.03797-23 38483478 PMC10986474

[36] Holtappels D, Alfenas-Zerbini P, Koskella B. 2023. Drivers and consequences of bacteriophage host range. FEMS Microbiol Rev 47:fuad038. doi:10.1093/femsre/fuad038 37422441

[37] Petty NK, Foulds IJ, Pradel E, Ewbank JJ, Salmond GPC. 2006. A generalized transducing phage (phiIF3) for the genomically sequenced Serratia marcescens strain Db11: a tool for functional genomics of an opportunistic human pathogen. Microbiology (Reading) 152:1701–1708. doi:10.1099/mic.0.28712-0 16735733

[38] Vieira MS, Duarte da Silva J, Ferro CG, Cunha PC, Vidigal PMP, Canêdo da Silva C, Oliveira de Paula S, Dias RS. 2021. A highly specific Serratia-infecting T7-like phage inhibits biofilm formation in two different genera of the Enterobacteriaceae family. Res Microbiol 172:103869. doi:10.1016/j.resmic.2021.103869 34333135

[39] Shymialevich D, Błażejak S, Średnicka P, Cieślak H, Ostrowska A, Sokołowska B, Wójcicki M. 2024. Biological characterization and genomic analysis of three novel Serratia- and Enterobacter-specific virulent phages. Int J Mol Sci 25:5944. doi:10.3390/ijms25115944 38892136 PMC11172527

[40] Fernandes S, São-José C. 2016. More than a hole: the holin lethal function may be required to fully sensitize bacteria to the lytic action of canonical endolysins. Mol Microbiol 102:92–106. doi:10.1111/mmi.13448 27328857

[41] Cahill J, Young R. 2019. Phage lysis: multiple genes for multiple barriers. Adv Virus Res 103:33–70. doi:10.1016/bs.aivir.2018.09.003 30635077 PMC6733033

[42] Fekrirad Z, Kashef N, Arefian E. 2019. Photodynamic inactivation diminishes quorum sensing-mediated virulence factor production and biofilm formation of Serratia marcescens. World J Microbiol Biotechnol 35:191. doi:10.1007/s11274-019-2768-9 31768723

[43] Flemming H-C, Wingender J. 2010. The biofilm matrix. Nat Rev Microbiol 8:623–633. doi:10.1038/nrmicro2415 20676145

[44] Ramanathan S, Ravindran D, Arunachalam K, Arumugam VR. 2018. Inhibition of quorum sensing-dependent biofilm and virulence genes expression in environmental pathogen Serratia marcescens by petroselinic acid. Antonie Van Leeuwenhoek 111:501–515. doi:10.1007/s10482-017-0971-y 29101490

[45] Cornelissen A, Ceyssens P-J, T’Syen J, Van Praet H, Noben J-P, Shaburova OV, Krylov VN, Volckaert G, Lavigne R. 2011. The T7-related Pseudomonas putida phage φ15 displays virion-associated biofilm degradation properties. PLoS One 6:e18597. doi:10.1371/journal.pone.0018597 21526174 PMC3079711

